# Basic Medical Training for Refugees via Collaborative Blended Learning: Quasi-Experimental Design

**DOI:** 10.2196/22345

**Published:** 2021-03-24

**Authors:** Thibault Lovey, Paul O'Keeffe, Ianis Petignat

**Affiliations:** 1 Department of Public Health & Global Health Division of Infectious Diseases, Epidemiology, Biostatistics and Prevention Institute University of Zurich Zurich Switzerland; 2 InZone University of Geneva Geneva Switzerland; 3 Division of Infectious Diseases Hôpitaux Universitaires de Genève Geneva Switzerland

**Keywords:** refugees, blended learning, basic medical training, higher education in emergencies, innovation, mobile phone

## Abstract

**Background:**

Globally, there is an excess of 68.5 million people who have been forced to leave their homes and seek sanctuary elsewhere because of poverty, persecution, conflict, violence, and human rights violations. Although international humanitarian responses usually focus on ensuring that the basic needs of these people are being met, there is growing attention on the role that development-oriented interventions can play in the longer term. Higher education in a refugee context is one such intervention that can equip refugees with the knowledge and skills they need to serve their communities and move forward.

**Objective:**

This study aims to evaluate the outcomes and effectiveness of the University of Geneva InZone-Raft Basic Medical Training Course in the Kakuma refugee camp in Kenya compared with a previous incarnation of the same course in the Dadaab refugee camp in Kenya.

**Methods:**

We used a quasi-experimental design to compare the posttest scores of both inequivalent student groups: control group (n=18) and intervention group (n=16). Factors that influenced refugee students’ knowledge acquisition, the amount of knowledge they acquired, and their academic outcomes were assessed, and the pedagogical evolution of the project is presented.

**Results:**

We found that the Kakuma intervention course yielded better outcomes and was more effective in terms of learning than the Dadaab control course. Of the 16 students who took part in the intervention course, 10 (63%) completed the program successfully and received accreditation from the University of Geneva. We observed that they received new knowledge well and scored higher on all learning modalities than those in the control course. Comparison of written and oral examinations between the courses showed statistical significance for the intervention group in written and oral exams (two-tailed: *P*=.006 and *P*=.05; one-tailed: *P*=.003 and *P*=.03, respectively). The Kakuma course was not effective in addressing electricity and internet access problems, nor in reducing the challenge of tight deadlines in the syllabus. Pedagogical adjustments to the intervention course improved student involvement, with higher participation rates in quizzes (10/11, 91%), and overall satisfaction and learning.

**Conclusions:**

The intervention group—with an improved mode of delivery, better contextualized content, and further interaction—reached a higher level of medical knowledge acquisition and developed more complex questions on medical topics than the control group. The positive outcome of this project shows that given the right resources and support, refugees can contribute to the improvement and development of health care in their communities. Nonetheless, a more focused effort is necessary to meet the educational needs of refugee learners and better understand their living conditions.

## Introduction

### Higher Education in Refugee Contexts

Academic discourse on the role of higher education has long centered on liberatory or functional objectives seen through humanistic or utilitarian lenses. Regardless of one’s ideological stance on these matters, most academics agree that the mission of higher education is multifaceted [1] and hinges on 3 important goals: delivering education, generating new knowledge, and engaging with society [2]. Through established pedagogical systems and social institutions, human progress has harnessed education to clear a pathway for those who participate within its confines to better understand, learn, and succeed.

Although all 3 goals of higher education play a critical role in constructing and understanding the world around us, the *society engagement* role is the engine in which we instrumentalize, form, govern, interact, and negotiate with the society or community and the wider world in which we exist. Whether economic, political, or social, societies in which higher education and social engagement are a priority are generally held to be successful and desirable places to live in [3]. In societies in which they are absent or lacking, there are many negative social implications, not just affecting theoretical future human progress but also real life here and now.

Emergencies, fragile states, poverty-stricken communities and refugee contexts are all places where higher education is absent, lacking, underresourced, and underresearched. Higher education in refugee contexts is a relatively new academic field of pedagogical scholarship that focuses on the specifics and outcomes of higher education in refugee communities. Growing out of sustained global efforts in the latter half of the 20th century and the early part of the 21st century to use primary and secondary level education as a driver for development, higher education in refugee contexts has been primarily rooted within the global forced migration management system as it attempts to contend with ever-growing populations of displaced and desperate people. As an increasing number of people enter the global forced migration system and/or come up through its management infrastructure, higher education and its application for human development is gaining traction as a key solution that may provide hope for averting or deepening humanitarian crises. Global initiatives such as the Sustainable Development Goals (SDGs) for 2030 have firmly positioned higher education as a policy direction [4]; however, little attention has been paid to developing appropriate pedagogical models that work for most of the globally displaced—refugees stuck in underresourced and overburdened refugee camps in poor and fragile states.

### The Reality of the Globally Disposed

The global refugee and displaced population is currently in excess of 68.5 million people, with one person forcibly displaced every second [5]. Most (85%) of the globally displaced live in low- and middle-income countries, with little access to basic services such as education and health care [6]. Although this unprecedented number grows daily, the very nature of the conflicts and abuses driving these figures often results in refugee populations spending excessive amounts of time—5 years or more—in exile because of the protracted nature of the situations in which they find themselves [7]. According to the United Nations High Commissioner for Refugees (UNHCR), nearly 16 million people (78% of the total world refugee population) found themselves in protracted refugee situations in 2019—a 12% increase in figures from the previous year [8]. Through no fault of their own, more young people are finding themselves stuck in limbo-like situations in refugee camps close to conflict zones for longer. Their dwindling life prospects, economic disempowerment, vulnerability to health crises, and many other social and political ills have given them less of a chance to live the kind of lives they frequently glimpse at through foreign movies and websites on their smartphones.

Recognizing the limiting life opportunities offered by refugee camps, the desire of young people to advance their own lives, and indeed the necessity of finding durable solutions to the disruption that displacement brings, humanitarian responses have begun to seek more sustainable approaches. High on the international agenda is the move beyond immediate humanitarian interventions toward development as a goal in itself. The 2030 Agenda for Sustainable Development, encapsulated by 17 SDGs, or a “blueprint for peace and prosperity,” enunciated this new direction by stating that the recognition “that ending poverty and other deprivations must go hand-in-hand with strategies that improve health and education, reduce inequality, and spur economic growth” [9].

### Development Through Education or the Status Quo?

With greater emphasis being placed on role development in finding durable solutions to displacement problems, the education for development conversation has moved on from the provision of primary and secondary education to include the role that higher education can play in development. The call for “lifelong learning opportunities for all” in SDG 4 [9] has further added power to the argument that higher education can provide a development solution for displaced populations.

Furthermore, higher education lies at the heart of the resilience and empowerment discourse popular in forced migration academia, policies, and practice. Education is often touted as providing a sense of purpose amid the uprootedness of refugee status and life constraints in refugee camps [10,11]. It is championed for playing a vital role in facilitating endurance and transitions for refugees by providing them with the skills they need to increase their social capital and ability to adapt to different and challenging contexts [12-15].

However, despite all the praise, the academic inquiry into the role of higher education in refugee contexts is not always positive. Critical academic approaches that take a more liberatory focus question the impact of higher education in propagating a system that keeps refugees contained and oppressed. In addition, the evolution of higher education in refugee contexts is often characterized as a neocolonial enterprise. Critics point to most of the teaching and learning programs accessible to refugees coming from Western institutions [16], being noncontextualized for refugee contexts and delivered through Western perspectives [17] via Western-centric web-based education platforms [18].

### Accessing Higher Education

Although there may be many questions left to answer about the purpose of higher education in refugee contexts and its intersection with authority, power, and control, there is the certainty that the pedagogical conditions that students in refugee camps have to contend with do not match with the condition of students elsewhere [19]. Some of the major obstacles they face in participating in higher education programs include the physical space where they find themselves, security issues that emanate from states of fragility, and major resource deficits that they experience [20]. Traditional *brick and mortar* higher education institutions tend to be in urban areas and/or require infrastructure that is not conducive to refugee camps and are often out of reach of refugees. Advancements in communication technology and web-based learning platforms have begun to address these shortcomings by paving the way for other possible solutions for bringing higher education to remote and inhospitable refugee camps. Massive open online courses (MOOCs; although problematic in themselves as they require technical capacity and tend to be designed with nonrefugee audiences in mind) and other web-based platforms—provided there is a technical capacity in place—can offer learners in refugee camps higher education opportunities they otherwise would not have. However, opportunities aside, to be successful, refugee students often require fortified scaffolding to support their learning [20].

In a synthesis of the literature, Bawa has found that web-based learning programs have a dropout rate of 40% to 80% in high resource contexts [21]. In situations such as refugee camps, where electricity is not always guaranteed, and computers and the internet are rare and expensive commodities, web-based learning may be a less than appropriate option [19]. On the other hand, blended learning approaches may offer the necessary support to ensure success for refugee learners. Although research and the literature on the benefits of blended learning in refugee camps are hard to come by, a pedagogical model, which has been found to be successful (and central to this case study), is the InZone collaborative blended learning model. This model has been found to achieve a course completion rate of 82.6% for refugee students who participated in higher education courses in Kakuma and Azraq refugee camps between 2017 and 2018 [22]. The model is presented below.

### Kakuma Refugee Camp

Located in the remote and arid Turkana state in North-Western Kenya, the Kakuma refugee camp is home to over 197,341 refugees from around 20 different nationalities, representing 39% of all registered refugees in Kenya [23]. Nationalities include South Sudanese, Somalians, Congolese, Ethiopians, Burundians, Sudanese, Ugandans, Rwandans, and Eritreans [24]. The camp was created in 1992 in response to the civil war in Sudan, which saw 20,000 young boys (the *lost boys of Sudan*, seeking sanctuary in neighboring countries [24]). Climate conditions are extreme. Temperatures routinely reach 38° C during the hot season, and rainfall is sparse. The camp is administered by the UNHCR and the Kenyan state and follows a containment policy with strict restrictions on movement in, out of, and around the camp. It is divided into 2 operative areas: Kakuma refugee camp and Kalobeyei integrated settlement. Although relatively safe compared with other refugee camps in the region, there are frequent security breaches [25]. Health care in Kakuma is serviced by 4 small clinics and Kakuma Mission Hospital, which has 62 beds [26]; however, overcrowding, inadequate sanitation, and poor access to these basic health services make Kakuma a dangerous place to live [27].

### InZone-Raft Basic Medical Training Course

**Figure 1:**
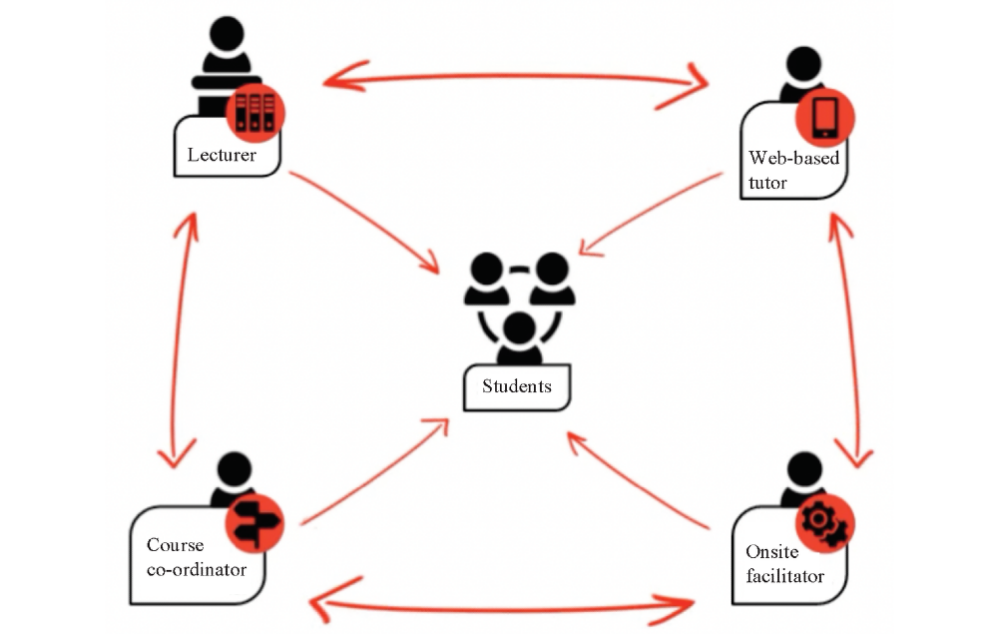
InZone collaborative learning ecosystem.

InZone, an academic and humanitarian program at the University of Geneva, has been active in the Kakuma refugee camp for a decade, where it enables accredited higher education courses via a collaborative blended learning ecosystem. InZone has a center (the learning hub) in Kakuma where students come to access computers and Wi-Fi, meet with each other, and connect to web-based courses from the University of Geneva. The InZone learning ecosystem, which scaffolds the students, takes a student-centered approach where learning materials and lectures are delivered through the web and facilitated by a refugee management team in a refugee camp. Students receive pedagogical support from web-based tutors from the University of Geneva, who *meet* the students through online tutorials, via WhatsApp instant messaging, for a designated amount of time each week throughout the course. Students are also supported by trained onsite facilitators in the learning hub, who assist the tutors in managing the class and the learning process. The learning ecosystem is overseen by a course co-ordinator who helps ensure the overall smooth running of the course. Figure 1 depicts the InZone collaborative learning ecosystem, with the following explaining the role of each of its actors.

### Key Actor Roles and Responsibilities in the Collaborative Learning Ecosystem

1. The lecturer delivers the course material over a learning platform (eg, via MOOCs freely available on Coursera or EdX), encourages the generation of new knowledge, and evaluates the students’ learning. In the ecosystem, the delivery of knowledge via a web-based platform enables the transmission of information to the students, who through discussions, group work, and so on acquire and develop new knowledge.
2. The web-based tutor is a subject matter expert or peer with a more advanced level of subject knowledge. The tutor plays a pedagogical role in this collaborative learning ecosystem by *meeting* the students regularly over an information communication technology platform (eg, WhatsApp) to stimulate new knowledge acquisition, discuss the student’s progress, and offer advice on being a successful learner. The web-based tutor also travels to the camp to meet the students in person and deliver face-to-face classes toward the end of the course.
3. The onsite facilitator provides onsite technical and guiding support to learners, helping them access the learning platform on location and navigate the physical learning space. The onsite facilitator is a critical contact point in the educational relationship among students and other members of the collaborative learning ecosystem as they are in frequent physical contact with the students.
4. The course co-ordinator has the overall responsibility for the day-to-day running of the course and liaises with other members of the learning ecosystem to ensure a smooth operation.
5. The student is the focal point of the learning ecosystem. This means that they are central to the collaborative learning model, and the entire learning ecosystem is designed to support their optimal learning by meeting their educational needs and promoting progressive learner autonomy [22].

In 2017, following the successful implementation of a basic medical training course in the Dadaab refugee camp in Northern Kenya, InZone was approached by Kakuma refugee camp authorities to develop a health care course for residents in the camp. The impetus for developing the course was the hope that locally trained refugees could partially fill the lack of staff in the health care sector in the camp [28]. To do this, InZone connected with the Raft telemedicine network at the University of Geneva and created a basic medical training course that could be enabled through InZone’s collaborative learning ecosystem in the camp.

Using lessons learned from a similar medical education program in Dadaab, the basic medical training course aimed to contextualize the curriculum, use a pedagogical methodology that best suits students’ needs, and ultimately prepare refugees to integrate into the health care services in the camp.

### Research on Higher Education in Refugee Contexts

As mentioned earlier, higher education in refugee contexts is an emerging field of study, with little literature covering medical pedagogy in refugee camps. To the best of the authors’ knowledge, little research has been carried out into which pedagogies would best satisfy the needs of refugee learners and the impact medical higher education can have on their communities. This study aims to add empirical evidence to this emerging field of study by evaluating the InZone-Raft basic medical training course across the 2 locations.

### Research Objectives

The primary objective of this research project is to compare the grades obtained by students in the Kakuma intervention course with the grades of the students in the Dadaab control course. This objective evaluates whether the students following a newer educational method employed in Kakuma would have higher scores in learning modalities (written and oral) than the control group in Dadaab, who followed an older educational model.

The second objective of this research is to evaluate the effectiveness of the new education model by quantifying the amount of knowledge acquired in the 2 groups. It was postulated that the Kakuma group would show an improvement in their acquisition of basic knowledge throughout the course compared with the control group in Dadaab.

The third objective of this research is to identify the confounding variables that influenced the final grades and thus the acquisition of new knowledge. It was assumed that external factors impact the acquisition of knowledge, particularly in a crisis context, and that these factors are common to the Kakuma and Dadaab cohorts.

### Research Questions

1. Did students who applied the newer contextualized blended learning methodology in the Kakuma intervention course obtain better posttest results than students who applied the traditional methodology in the Dadaab control course?
2. Was the education model used in the intervention course more effective in producing better periodical knowledge acquisition at set points throughout the course than the education model used in the control course?
3. What confounding variables may have influenced the student’s posttest results in both groups?

## Methods

### Study Design

Owing to ethical considerations, it was not possible to divide a cohort of refugee students for the purpose of this research. Consequently, randomization was not possible, and the study was conducted as a quasi-experimental study. The control group was nonequivalent and recruited from another classroom that was already following the older teaching model at the time this study began.

Refugee camps challenged the practicality of the study, as certain external factors could not be controlled. There are numerous barriers related to access to education that are present in refugee contexts and are therefore unavoidable. In this specific environment, the quasi-experimental design has the advantage of providing higher external validity by allowing the experiment to be implemented directly without the need for artificial modifications.

### Inclusion Criteria and Sample Size

Participants were recruited over 2 months, from August to September 2018, in the Kakuma refugee camp through the InZone selection process. To be admitted to the course, the applicant had to be a resident in the Kakuma refugee camp or Kalobeyei integrated settlement (a nearby refugee–host community settlement), own a high school diploma, satisfy the proficiency requirements for the English language with a C grade in the Kenyan high school state exam or equivalent, and write a motivation essay (Multimedia Appendix 1).

Functions for power analysis were used to evaluate the appropriate size of the intervention group [29]. The parameters were set to a significance level of .05, a power of .8, with a control group of 18 participants and an expected effect size of 1.2. The formula estimated the minimum sample size required for the two-tailed and one-tailed *t* tests of 9 and 6 students, respectively. With an estimated dropout rate similar to that of the control group, at 33% (9/27), the recruitment was set with a total of 16 students in the intervention group.

### Intervention

The basic medical training course consisted of 3 modules. Module 1 consists of an introduction to the major organ systems of the human body. It covers the physiology and anatomy of 13 organ systems. Module 2 is more clinically oriented and teaches basic illness physiopathology relevant to medical conditions in sub-Saharan Africa. Module 3 is a case-based learning unit in which students investigate the medical conditions of 9 patients. The content of the modules was discussed and evaluated by a multidisciplinary professional team composed of physicians, a pedagogue, and a sociologist to ensure high quality of content relevant to the refugee situation and consistent with the level of a higher education course.

To avoid the additional costs and difficulties of sending teaching materials to refugees and ensure almost instantaneous deployment of the course, all materials were available on the web at the University of Geneva’s Moodle learning platform (Moodle Pty Ltd) [30] in which the students had access to 13 units divided by organ system. Each unit was built identically with the corresponding Khan Academy videos (Khan Academy Ltd) [31], chapters from the OpenStax e-book *Anatomy and Physiology* [32], various additional documents to better strengthen their understanding of the topic, and a list of key concepts to be mastered for their final evaluation (Multimedia Appendix 2).

Computers and free Wi-Fi were accessible in the learning hub for the students to access at designated time slots throughout the course to ensure equitable accessibility among all course participants. Transport and meals at the hub were subsidized by course funds for the same reasons. Huawei Media Pad T3 10 tablets were also allocated to the students so that they could access preloaded learning materials offline outside of the allocated access times at the learning hub. A student was appointed as an onsite facilitator to assist in managing the day-to-day activities of the class and assist web-based tutors with lesson co-ordination.

At designated times each week, 2 medical students from the University of Geneva tutored the students via WhatsApp using structured web-based tutorials. These tutorials involved the presentation of questions and discussion points on a WhatsApp forum at the start of each week. Students and tutors later synchronously and asynchronously discussed these points and questions throughout the week. On average, for module 1, one system was studied each week with planning adjusted according to any difficulties experienced by the students (eg, flooding hindering students’ travel to the learning hub).

The procedure for the control group in the Dadaab refugee camp was identical except that tablets and Khan Academy videos were not provided to students.

### Sampling

The study received 45 applications to take part in the new education model in the Kakuma refugee camp. Following a selection procedure carried out by InZone, a cohort of 16 students was recruited to participate in the InZone-Raft basic medical training course, which ran from October 2018 to February 2020. They took part in module 1 of the course from October 2018 to February 2019.

Out of the 16 students who initially started the course, 12 completed it, 11 took a final written and oral exam, and 10 passed it. The reasons for dropping out of the course were *family problems* (n=2), moving out of the refugee camp (n=1), attending an alternative training course (n=1), and lack of commitment because of relocation (n=1). A total of 11 students were included in the intervention group for the statistical analysis, as they presented themselves in the final oral and written exam (Multimedia Appendix 3).

The control group consisted of 18 students from the cohort who followed the previous education model from February 2017 to February 2018 in the Dadaab refugee camp. Of the 27 students initially recruited, 18 completed the course and were included in the statistical analysis. Reasons for dropping out included relocation of non-Somali refugees to Kakuma camp, return to Somalia, relocation to other countries, and family problems [33] (Multimedia Appendix 3).

Demographic data (age, sex, and country of origin) and educational attainment data were collected from all participants during the application process.

### Measures and Instruments

The objectives of this quasi-experimental study focus on 3 particular aspects of module 1 that need to be measured in both groups: (1) posttest results, (2) knowledge acquisition, and (3) confounding variables.

#### Posttest Results

To ensure adequate measurement of student performance at the end of the first module, tutors traveled to the respective refugee camp to provide face-to-face revision classes for 2 days and a day of summative evaluation, including a supervised written exam and an oral exam. The written examination consisted of 53 multiple-choice questions and 18 development questions. The oral examination consisted of 6 questions related to the organ system described in a clinical situation and 2 additional questions related to a different system. The content of the written and oral examinations was discussed with the multidisciplinary team to ensure it was representative of all the topics covered in module 1, thus guaranteeing an adequate balance of questions.

Several methods have been used to ensure the highest possible reliability in the measurement of student knowledge. The written examination used multiple-choice questions to ensure impartiality and was marked anonymously by 2 different experts, and the marks were then cross-checked. The oral examinations used a standardized assessment grid, and student performance was assessed by 3 experts who were physically present at the assessment (written exam: Cronbach α=.92, 95% CI 0.86-0.98; oral exam: Cronbach α=.92, 95% CI 0.86-0.98) [34].

Quantitative data on written and oral examination results, final grades, and critical thinking questions were collected directly from the respective teachers for both cohorts at the end of module 1.

#### Knowledge Acquisition

Quizzes were used to assess the students’ progression at regular intervals throughout the course (at the end of each unit of study). These formative evaluations were divided into 3 levels of difficulty and could be repeated, if desired; a multidisciplinary team designed them to ensure that it assesses the acquisition of the key elements of each organ system addressed.

To assess student satisfaction and give suggestions for improvements, a web-based feedback survey was disseminated to the students at the end of each study unit and at the end of each module.

The quiz results and feedback for each unit were extracted from Moodle in the csv (comma-separated values) format. The module 1 course feedback was sent after the final exams to the students who completed the course via Google forms and then exported to Microsoft Excel.

#### Confounding Variables

To ensure sufficient internal validity, variables that could influence posttest results such as average quiz scores, Moodle logs, number of WhatsApp messages, time used to complete the written examination, number of hours of study per week, previous medical experience, and gender were taken into account in the analysis and interpretation of total scores.

For the activity on the students’ Moodle platform, the logs were extracted from Moodle for each student. The WhatsApp discussions between students were extracted directly from the application as a text file. The collection of Moodle logs, WhatsApp discussions, and critical thinking questions could only be obtained from the Kakuma cohort for technical reasons.

### Statistical Analysis

To protect the anonymity of the students, all names were coded before performing the statistical analysis. All statistical tests used an α of .05 as the significance level. R Studio [35] was used as the main part of the analysis.

#### Posttest Results

A descriptive analysis was carried out for the results of the written and oral examinations, the final score, and the quizzes of module 1 [34] to evaluate the results of both groups. The mean and median were used as central indicators of dispersion and expressed with a CI and a minimum and maximum value to assess the range.

To demonstrate whether the students who participated in the new method performed better than the control students, a posttest analysis was performed to assess the impact of the intervention, comparing the average between the 2 groups for the written exam, the oral exam, the final score, and the specific quiz questions with the overall statistics [36]. A one-tailed and two-tailed Student *t* test was used, and the nonparametric Wilcoxon signed-rank test was used when the distribution of scores was not normal.

#### Knowledge Acquisition

Only the first attempt was taken into account for the individual calculation of the average of each quiz. In addition, before the calculation of the individual average in the quiz of module 1, the 2 most extreme values of the individual quizzes were removed [37].

Spearman correlations were used to look for patterns between the quizzes and the final score to determine whether a method was more effective in producing knowledge acquisition throughout the course.

#### Confounding Variables

A WhatsApp discussion analysis [38] and text mining and sentiment analysis on WhatsApp text were performed to assess confounding variables that may influence student scores [39-42]. The student feedback was also evaluated [43].

Multiple regression analyses—with robust methods and variable transformations to ensure normality—were used to assess any confounding variables (including average results of the quizzes, Moodle logs, the number of WhatsApp messages, the time they used to complete the written exam, the number of hours per week they studied, their previous experience in medicine, and their gender) that might influence the final score in both groups [44-48].

### Ethical Considerations

This study was approved by the Swiss Ethics Committee on research involving humans. The protocol number assigned was 2017-00632. Participants were informed of the purpose of the research, the methods used, and that enrollment, successful completion, or withdrawal from this program did not affect any eventual resettlement or repatriation process.

Participation was voluntary, and all the participants provided written informed consent. The *WhatsApp Forum Guidelines for InZone-RAFT Basic Medical Training* code of conduct was also accepted and signed by all students before using the WhatsApp group (Multimedia Appendix 1). This study included qualitative data from a small sample of a vulnerable population. These data cannot be released to the public because of the potential risk of identifying vulnerable refugees. Special requests for access to the data were evaluated in collaboration with the Ethics Commission of the University of Geneva.

## Results

### Sociodemographic Characteristics

In the control and intervention groups, women accounted for 27% (control: 5/18; intervention: 3/11) of the total students. The country-of-origin data varied in both groups. In the intervention group (Kakuma), the distribution of country of origin was diverse among the students, with 18% (2/11) from Burundi, 27% (3/11) from the Democratic Republic of Congo, 27% (3/11) from Southern Sudan, 9% (1/11) from Sudan, 9% (1/11) from Ethiopia, and 9% (1/11) from Somalia. In the control group, the representation was narrower, with 94% (17/18) from Somalia and 6% (1/18) from the Democratic Republic of Congo. Prior knowledge or experience in health-related areas was present in 36% (4/11) of the intervention group and 39% (7/18) of the control group. Age distribution could not be calculated as, for sociocultural and political reasons, dates of birth are not always recorded by families or they are artificially generated by the asylum-granting authorities when individuals apply for refugee status (Multimedia Appendix 3).

The homogeneity test concerning the sociodemographic characteristics of the 2 groups showed no significant differences and therefore were both considered homogeneous.

### Posttest Results

For the written exam, with a possible total of 75 points, we obtained a mean score of 38 (SD 14; 51%) points, a median score of 35 (46%) points with a maximum score of 60 (80%) points, and a minimum score of 13 (17%) points. A question in the exam reached a perfect score (Q19), and 4 questions (Q28, Q33, Q43, and Q45) obtained a percentage of correct answers lower than 20% (2/11). For the oral exam, with a possible total of 20 points, we obtained a mean score of 13 (SD 5.5; 67%) points, a median score of 14 (70%) points with 1 perfect score, and a lower score at 2 (10%) points, which represents a question answered correctly. For critical thinking questions, with a possible total of 8 points, we obtained a mean score of 4 (SD 2.6; 53%) points, a median score of 4 (50%) points, 1 perfect score, and a minimum score of 1 (13%) point. If we combined the oral and written examinations by weighting the results by half between oral and written examinations, the mean percentage was 59% (SD 21.9) and the median percentage was 56% (range 14%-88%). For the Dadaab cohort of students studying the same course (control group), the mean percentage was 41%, median was 40%, maximum grade was 71%, and minimum grade was 15% (absolute numbers were not available; Table 1). We noted a significant difference between the previous cohort and this cohort in the total score (two-tailed: *P*=.008; one-tailed: *P*=.004*)*. The comparison between the Dadaab and the Kakuma cohorts’ written and oral examinations showed statistical significance for the intervention group in the written and oral exams (two-tailed: *P*=.006 and *P*=.05; one-tailed: *P*=.003 and *P*=.03, respectively), as shown in Table 1. Comparing the 5 identical developmental questions on the written test between the Dadaab and Kakuma cohorts yields a significant difference in means for this year group (two-tailed: *P*=.03; one-tailed: *P*=.02).

**Table 1:** Results by evaluation type for intervention and control groups.

Evaluation type			Mean								*P* value					Median									Range (%)			
			Mean score (SD)		Percentage			95% CI			Two-tailed			One-tailed				Median score			Percentage		95% CI					
**Experimental group**																												
	Written exam (total possible score: 75 points)	38 (14)		51			38-63			.006^a^			.003^a^				35			46				36-75			17-80	
	Oral exam (total possible score: 20 points)	13 (5.5)		67			46-56			.05^b^			.03^b^				14			70				40-95			10-100	
	Final results	—^c^ (21.9		59^d^		44-74			.008^e^			.004^e^			—^c^				56^d^			43-88				14-88		
	Critical thinking (total possible score 8 points)	4 (2.6)		53			32-75			N/A^f^			N/A				4			50				13-88			13-100	
	Quiz results (total possible score: 10 points)	5 (1.3)		48			40-56			N/A			N/A				5			46				38-66			29-69	
**Control group**																												
	Written exam	—^g^		30			23-38			N/A			N/A				—^g^			31				22-39			0-56	
	Oral exam	—^g^		51			43-59			N/A			N/A				—^g^			46				40-53			30-93	
	Final results	—^g^		41			34-48			N/A			N/A				—^g^			40				36-45			15-71	

^a^Comparison of written exam mean scores between groups (Student *t* test).

^b^Comparison of oral exam means between groups (Wilcoxon signed-rank test).

^c^Not available.

^d^Calculated by combining written and oral examination results weighted by half.

^e^Comparison of final results mean scores between groups (Wilcoxon signed-rank test).

^f^N/A: not applicable.

^g^Absolute numbers not provided by teachers.

### Knowledge Acquisition

The intervention cohort’s average for the 19 quizzes for module 1, with a possible total of 10 points each, had a mean score of 5 (48%) points, a median score of 5 (46%) points, a maximum score of 7 (69%) points, and a minimum score of 3 points (29%; Table 1). The median participation rate was 91% (10/11) of students for the cohort. There was a positive correlation between the quizzes and written scores (ρ=0.93; *P*<.001) and an even stronger correlation with the total score (ρ=0.94; *P*<.001)*.* A total of 25 quiz questions used for the control cohort were reused for the intervention cohort, and it was found that 4 quiz questions had a statistically significant higher score for last year’s group and 2 for this year’s group.

### Confounding Variables

#### WhatsApp Forum

On the WhatsApp discussion forum, which comprised 21 members, the chat started on October 22, 2018, at 9:20 AM. In total, 677 messages were sent to the participants (including 148 media messages). The most active day on the forum was October 28, with 23 messages sent, the most popular day for sending messages was Saturday (n=112), and the preferred time for texting was 11 AM (n=54; Multimedia Appendix 4).

The most active students were S10 (n=204), followed by S5 (n=57) and S12 (n=44), whereas those who answered the tutors’ questions the most were S10 (n=44), followed by S12 (n=12) and S5 (n=9). Sentiment analysis of 10 different feelings was performed using text mining techniques, and the most representative sentiment was *positive* (n=516), followed by *trust* (n=344), and the least representative was *disgust* (n=39; Multimedia Appendix 5).

#### Feedback

In the feedback form, we found that the main source of learning materials was the Khan Academy videos (n=11), followed by the WhatsApp group forum (n=4).

Students accessed the InZone learning hub 5 times per week (median) and studied 20 hours (median). In general, the feedback was positive for the 11 students who finished module 1. The level of the content (8/11, 73%), amount of information to learn (8/11, 73%), and the level of English (9/11, 82%) were all adequate for the students. An exception was that the students felt the time available to study was too short for 7 (64%) of them. The main obstacles highlighted for the course were access to electricity (8/11, 73%) *every day*, internet access (7/11, 64%) *every day*, and access/transport to the hub (6/11, 55%) *every day*. Cultural barriers (10/11, 91%) *never*, lack of teaching support (6/11, 55%) *never*, lack of prior education (5/11, 45%) *never*, lack of space to study (8/11, 73%) *never*, and money (7/11, 64%) *once a month* were not identified as potential barriers. Personal issues were more variable among the students *rarely* (mode).

#### Multiple Linear Regression

Multiple linear regression was calculated to predict the final score in the intervention group based on the average of the quizzes, Moodle logs, the number of WhatsApp messages, the time they used to complete the written exam, the number of hours per week they studied, their previous experience in medicine, and their gender. A significant regression model was found (*F*_3,7_=23.89; *P*<.001), with an *R*^2^ adjusted to 0.87. The participants’ predicted final score was equal to 1.01−0.19 (exam time)+0.28 (previous experience)−0.40(gender), where exam time was measured in hours, previous experience was coded as 1=yes, 0=no, and gender as 1=woman, 0=man (Table 2). The participants’ final score diminished by 19% for each extra hour, previous knowledge increased the final score by 30%, and women scored on average 40% less than men if all other variables remained constant. Regression in control groups holds the same conclusion and therefore is not detailed.

**Table 2:** Multiple linear regression to predict the final score in the intervention group.

Predictors	Estimates	95% CI	*P* value
Intercept	1	0.68 to 1.33	<.001
Written exam time	−0.19	−0.33 to −0.04	.02
Previous experience	0.28	0.15 to 0.40	.001
Gender	−0.4	−0.54 to −0.27	<.001
Observations	11	N/A^a^	N/A
*R* ^2^	.91	0.84 to 0.98	N/A
*R*^2^ adjusted	.87	0.77 to 0.97	N/A

^a^N/A: not applicable.

## Discussion

### Posttest Comparison Between the Cohorts

To evaluate the Kakuma students’ learning throughout the course, we started by looking at their exams results. We grouped the cohort into 3 main groups: a group with 3 students who exceeded our expectations, a pre-eminent group that met the minimum conditions, and a student who was behind the others. The results follow a Gaussian distribution and reflect the teachers’ initial assessment of the student’s level. In addition, teachers were impressed by the complexity of the questions asked by the students during revision sessions. In their opinion, highly motivated students attained a level of medical knowledge comparable with the first-year class in a medical school in the West.

One of the main objectives of this study was to evaluate the effectiveness of the new education model. If we consider the average score for all students in both cohorts, we have a significant difference in both two-tailed and one-tailed results for the written exam, oral exam, and total score. Overall, we can say that the Kakuma cohort had a better overall grade, but a disparate comparison of exams is questionable. We cannot exclude that this intervention cohort’s written or oral exam was simpler than the control cohort in Dadaab.

As a result, we paid particular attention to specific questions in the Moodle quiz. Of 29 identical questions, 4 were significantly better for the control group and 2 for the intervention group. This result does not confirm our initial hypothesis that the intervention group scored better on average. However, on average, we had a double participation rate compared with the previous cohort, which can create a bias, as only the most motivated students from the control cohort participated in quizzes. Therefore, we looked further and considered the same questions in the written exam. There was a significant difference between the 2 scores in favor of the intervention group. In doing so, the inequity in the response rate is eliminated and ensures the same parameters. We interpret this improvement as emanating from the learning videos and interactive quizzes, making a difference to students’ knowledge acquisition. Khan Academy videos give the effect of a live explanation, and quizzes with progressive levels help motivate students to learn—as testified by a participation rate close to 100%.

### Improvement of Initial Knowledge Inside Groups

Determining whether the students had improved their initial medical knowledge during the course was difficult to assess. We inquired whether students had a background in the medical field before studying the course in the feedback form. Our analysis indicates that prior medical knowledge has a positive impact on the score, and a correlation was found between background knowledge and the final score in both groups. This could reveal 1 of 2 conditions: either students with previous health education learned something new or the group was too small to demonstrate a correlation. In either case, as *R*^2^ adjusted was large, the size of the cohort does not change the correlation strength. Similarly, we compared the average of the quiz with the final result, which revealed a strong correlation. In the model, 87.3% of the final score variability was expressed as the average of the quiz. The excellent results of quizzes and final exams prove that their new knowledge was well received. We also established correlations among the exam questions.

### Confounding Variables Regarding Final Scores

To identify any confounder that could enhance students’ total scores, we first considered participation in the WhatsApp forum. We found no direct correlation between the number of WhatsApp messages posted by individual students and their final scores. However, the top scorer, S12, was also the most active on the WhatsApp forum, which came second for him as a study resource. In any case, in this challenging environment, we understand that not everyone has an electronic device and that the smartphones used to access WhatsApp were probably shared among students. However, we are reassured that the WhatsApp group is an excellent cohesive tool that gives all equal opportunities.

Second, we looked at the students’ Moodle logs, which revealed no correlation. This reflects the basic activity of the Moodle platform but not engagement. With the new course organization focused on Khan Academy videos, the Moodle platform was relegated to be a tool where students could answer the quiz and evaluate their progress.

Finally, we noticed a significant negative correlation for gender, with women scoring 40% less than their male counterparts. Indeed, “traditional mechanisms of protection and social norms remain, but deviate substantially within the refugee context including the attitude and the perception of the ‘proper’ role of women. In some instances, this triggers a positive redefinition. In other cases, however, traditional mechanisms of protection and social norms remain in and this often results in stigmatization of the refugee woman by her community” [49].

With this in mind, we acknowledge that the lack of or reduced empowerment of female students negatively impacted their performance in the oral exam. This difference was not realized as strongly in the control group; the difference in score between males and females was 4.6% (*P*=.40) and not significant [33].

### Impact

Of all participants, 10 students received accreditation from the University of Geneva (6 European Credit Transfer System [ECTS] credits) for module 1 and 1 student received a certificate of participation. Receiving ECTS credits allows students to transfer these credits into other medical training programs. The course has been discussed with local universities, and we aim to develop cooperation and future education pathways to advance students’ studies.

One of the main problems identified with the Dadaab cohort project was the lack of critical thinking about the information they were learning. During the oral exam for the Kakuma cohort, we used a critical thinking question as a sensor to see if the students could apply what they learned to a new situation, in this case, a clinical problem. The results were presented as a bimodal distribution, which means that there is a clear separation of each student’s ability to process what they learned. Some were perfectly capable of criticizing what they learned and using the information in a different context. Others needed guidance to do so, but they could be taught and learned. However, in a different educational system where knowledge is imparted, it is difficult to adjust the study approach.

This course also shows that there is flexibility in the learning system. All content is accessible via the Moodle platform and the tablets provided to the students at the start of the course. It is possible to take the course in other difficult refugee contexts. One of the students, for example, had to leave the camp and continued to attend the course and participated in the WhatsApp forum remotely. New resources and technologies could be incorporated into the course to further improve accessibility, which can be challenging in a refugee camp environment.

Finally, students told us orally and in their written feedback about their hopes to have a positive impact on their communities’ health. They expressed their desire to improve the lives of the people around them by using their new knowledge. After a meeting with the International Rescue Committee (IRC), they confirmed that there is a need for more people to work in Kakuma’s health care system. Consequently, the IRC expressed a desire to hire medical students in the camp’s clinical facilities. In response, teachers have oriented the end of modules 2 and 3 to include some basic clinical gestures to help them achieve their objectives.

### Limitation and Future Direction

The dropout rate in the newer cohort in Kakuma (5/16, 31%) was similar to that in the control group (9/27, 33%). The main reason for dropping out given by the students was *personal reasons*. In our view, although personal reasons cannot be anticipated at the time of recruitment, we recommend more clarity to the students before enrollment on the commitment needed to finish the course successfully.

The main criticisms for module 1 in both cohorts were the amount of time students needed to study. The multiplicity of content and tight deadlines were challenging for students. Even by making some schedule adjustments, the course is very demanding, and in the unstable context of a refugee camp, it can become difficult for some students to keep up with the required pace. Access to electricity, the internet, and more generally to the learning hub remains to be a problem for students in Kakuma refugee camps. The use of tablets may have been a way to ameliorate these challenges, with all the materials preloaded on the tablets. Unfortunately, for various security-related reasons, students could not take them home with them to study in the evenings and weekend days (eg, carrying valuable technology in the camp puts the students at risk of being attacked and robbed).

As with all web-based and blended learning courses, maintaining motivation is a challenge students face, which affects course completion rates. The education model used in Kakuma attempted to address this problem with a more interactive course to promote greater student involvement. The change in strategy to include students in the decision-making process and the heightened role of the onsite facilitator appears to have had a positive impact on the students’ overall performance.

### Future Directions

Going forward, we recommend evaluating the long-term impact of the program. A longitudinal study following the alumni to measure their impact on their communities and the opportunities they received would be a valuable resource for the growing field of higher education in refugee contexts. Such a study would also help universities and other education providers operating in refugee spaces to refine their offers and better serve refugee students.

We also recommend that multiple courses are offered simultaneously and in several camps so that more refugees can attain the knowledge that they need to better ensure the health and well-being of their communities. Randomization would therefore be possible, thus ensuring greater internal validity. In addition, the introduction of a preliminary examination to assess the level of knowledge of course applicants more deeply would allow better organization of classes along capacity lines.

Finally, we suggest that any future courses for refugee learners consider the lived realities of refugee learners in refugee camps. Course content needs to reflect refugee needs and not be merely parachuted in via Western-centric digital modes. Blended learning will work if there are sufficient support and technological and pedagogical resources available for scaffold learning. To help achieve this, we recommend finding solutions so that students are safe and have greater access to technology. For example, giving them individual tablets at the start of the course and unlimited access to the learning hub could increase course participation and learning. In addition, contextualizing learning materials according to needs assessments and heightening the pedagogical and pastoral role of onsite facilitators will help to achieve better outcomes for all.

### Conclusions

The InZone-Raft project has provided refugees in Kakuma and Dadaab refugee camps with high-level basic medical training in 2 of the most challenging environments in the world. We found that by improving the mode of delivery, better contextualizing content, and promoting more interaction between the students and their teachers, the Kakuma student cohort reached a high level of medical knowledge and was able to develop complex questions on medical topics. Their results in quizzes and final exams of their course prove that their new knowledge was well received, and the education model was more efficient than its earlier incarnation in the Dadaab refugee camp.

As we move forward in developing and delivering medical courses, those of us working with refugee learners need to pay more attention to our students. Given the exponential growth of the globally displaced in recent years, it is evident that the demand far outweighs the supply. However, this cannot be an excuse to cut corners and implement courses that do not work in refugee contexts. Much more positive results are achieved when refugees are acknowledged, included, and provided with the resources they need.

The positive outcome of this project shows that given the right resources and support, refugees have the capacity to contribute to, improve, and develop health care in their communities. However, to achieve this, a more concerted effort needs to be made to meet the educational needs of refugee learners and better understand the space in which they live. Higher education for refugee context research is one of the keys to achieve this. By better understanding the pedagogical dynamics that operate in these spaces, we can move forward with successful programs such as the InZone-Raft basic medical training course in Kakuma refugee camps.

The basic medical training course has been the subject of many requests from potential students and health care workers in Kakuma, as there is a lack of adequate education programs available in the camp. If there is the will to embrace it at the forced migration management administration level, this medical course could allow refugees to integrate into the health services in the camp. The positive impact this would have on the health and well-being of refugees in Kakuma could serve as inspiration that refugees can be empowered through higher education to serve and lead their own communities. Therefore, we recommend that similar education programs be embraced and implemented in refugee camps globally.

## Appendix

Multimedia Appendix 1 InZone-Raft Basic Medical Training application form with an overview of the program, inclusion criteria, application form and motivational essay, and WhatsApp forum guidelines.

Multimedia Appendix 2 The syllabus of Module 1 of the Basic Medical Training with unit number, names of organ systems, references of videos of the Khan Academy, references of book chapters, absolute numbers of questions in quizzes, average of quizzes per level, and duration of each unit in weeks.

Multimedia Appendix 3 Complete characteristics of the intervention and control group participants including gender, prior knowledge, origins, and absolute frequency by group, mean, standard deviation, median, median absolute deviation, range, kurtosis, skew, and standard errors of the final scores broken down by gender and prior knowledge in both groups.

Multimedia Appendix 4 WhatsApp group analysis of the module 1 intervention cohort was performed using WhatsAnalyzer. It includes general statistics, messages sent per user, messages sent on average during module 1, messages sent on average per day, messages sent on average per hour, and the communication network between users.

Multimedia Appendix 5 Feelings were analyzed in the WhatsApp chat intervention by generating the global cloud, retrieving feeling words from the text, counting feeling words by category, printing the feeling plot, and creating a global text mining cloud. In addition, a graphical representation of the sentiment analysis is provided for 10 different sentiments in the WhatsApp intervention group.

## Abbreviations

ECTS: European Credit Transfer System
IRC: International Rescue Committee
MOOC: massive open online course
SDG: Sustainable Development Goal
UNHCR: United Nations High Commissioner for Refugees
