# Supplementary material for: Basic Medical Training for Refugees via Collaborative Blended Learning: Quasi-Experimental Design
Source: J Med Internet Res. 2021 Mar 24;23(3):e22345. doi: 10.2196/22345 (PMC8074855; doi:10.2196/22345)
Supplement: Multimedia Appendix 1 [file jmir_v23i3e22345_app1.pdf]

Multimedia Appendix 1. Application form and WhatsApp chart.

Organisation des Nations Unies pour l'éducation, la science et la culture

Chaire UNESCO en formation médicale numérique, Université de Genève

BASIC MEDICAL TRAINING FOR KAKUMA REFUGEES & HOST COMMUNITY YOUTH

PROGRAM DESCRIPTION

Overview

The following programme, offered by RAFT and InZone at the University of Geneva aims at providing healthcare workers of the Kakuma refugee camp with high-quality, certifying, medical tele-education. This program comprises an introduction to basic medical sciences, such as anatomy and physiology, but also key knowledge about the epidemiology, the diagnosis and the management of frequent and relevant medical conditions encountered in Sub-Saharan Africa.

The program is divided into two main blocks: Basic Medical Sciences and Human Pathology (which are further described below) for a total duration of 10 months (40 weeks, including examinations). The first block will cover key principles of biology, anatomy and physiology and the second block is more practical and oriented towards acquisition of knowledge on frequent and/or relevant medical conditions in Sub-Saharan Africa.

Student profile

This course is directed to highly-motivated high-school graduates with a minimum grade of C plain in the KCSCE or equivalent grade in a recognised secondary school leaving certificate, who would like to gain skills for an entry level job in the health sector or are pursuing a career in health science.

This is an independent training programme. Enrolment, successful completion or withdrawal from this programme has no impact on any potential resettlement or repatriation process.

Course leader:

Professor Antoine Geissbuhler, MD

Course support staff:

Paul O’Keeffe, PhD

Course organisation:

InZone - University of Geneva (Professor Dr. Barbara Moser-Mercer)

Required education level for entry into the program	This is a Higher education program; applicants must have completed secondary education. KCSE, minimum grade C plain
Age range	Youth (18-32)
Gender	We strive to achieve a balanced gender representation (Objective 65/35)
Host community inclusion	25% of study places; spaces not allocated to host community students will be allocated to refugee learners
Cost of program	This program comes at no cost to the student.
Course provider	Université de Genève – RAFT/InZone
Title of course	Basic Medical Training Course
Language of course	English
Course dates	October 3 2018 – February 28 2019
Number of students working hours hours/week	6-10
Learning Platform	University of Geneva - Moodle
Requirements	Proficiency in English (read and write)
Tutor support	On-site: 2 dedicated e-learning facilitators<br>On-line: 1 professional academic tutor
Certification	Certificate of completion; possibility to obtain academic credit
Number of students admitted for this course	15-20
Application documents	1. Application form (see below) filled in and signed<br>2. Secondary School Leaving Certificate (scan of original)
Application deadline	**11/10/ 2018**

Deposit on-site and send application documents to:	**Deposit at InZone Learning Hub Kakuma 2 (CTA)**

Registration form (Application deadline September 28, 2018)

Last name, first name	
Gender	
Date of birth	
Languages	
Secondary school leaving certificate delivered by:<br>Date of delivery :	
Number of higher education courses completed	

Please write 200 words about why you would want to take the Basic Medical Training Course and how you expect to be able to apply your knowledge and skills after completing this course<br>(You may use a separate piece of paper or create a new file, but you must label your file clearly with your full name and date of application)	

PLEASE DEPOSIT A COPY OF YOUR APPLICATION at the InZone Learning Hub Kakuma 2 (CTA Don Bosco)

Please copy electronically: Barbara.Moser@unige.ch , Paul.Okeeffe@unige.ch

Whatsapp Forum Guidelines for InZone –RAFT Basic Medical Training

1. This group is for discussions related to the InZone-Raft Basic Medical Training

2. The forum is primarily intended for students to discuss learning topics. Onsite facilitators, online tutors and management will also be present on the forum, but we strongly encourage all students to participate fully in the forum by initiating discussions and conversing with each other.

3. All discussions on the forum must relate to the course. Any non-relevant posts will be removed by the administrator.

4. Participants in the group are expected to act respectfully and professionally at all times.

5. Participants are reminded that inappropriate language, images or other media must be kept off the forum.

6. To the extent that is possible, participants should keep all discussions on the public forum, rather than private messaging, to ensure that everyone learns from all discussions.

7. If you change your number during the course, please inform the administrator (Paul from InZone) immediately, so he can add you to the forum.

8. The information discussed in the forum is intended for group members only. Please do not share the content with people from outside the course (unless agreed upon with InZone and MIT management.

9. It is important to remember that not all members of a Whatsapp group may have the same access to internet and other resources. Therefore, it is advised to keep the conversations in and around designated class time, where possible, as students will have access to Wi-Fi during this time and will not be over-burdened by using their own money to pay for credit.

10. If you have any grievances regarding the forum or participants in the forum please email

    Paul.Okeeffe@unige.ch outlining the issue.

.

Date　　　　　　　　　　　　　　　　　　　　　　Signature

____________________　　　　　　　　　　　　　____________________
