# Supplementary material for: Basic Medical Training for Refugees via Collaborative Blended Learning: Quasi-Experimental Design
Source: J Med Internet Res. 2021 Mar 24;23(3):e22345. doi: 10.2196/22345 (PMC8074855; doi:10.2196/22345)
Supplement: Multimedia Appendix 2 [file jmir_v23i3e22345_app2.pdf]

Multimedia Appendix 2. Module 1 basic medical training program, quizzes results and duration.

Unit [no.]	Organ system [name]	Khan academy® videos [title]	*Anatomy and Physiology*, book chapters [title]	Quizz questions [n]	Mean quizz [%]	Duration [week]
1	Cardiovascular	- Meet the heart!<br>- Flow through the heart<br>- Two circulations in the body<br>- Lub dub<br>- Layers of the heart<br>- Thermoregulation in the circulatory system<br>- Arteries vs. veins - what's the difference?<br>- Arteries, arterioles, venules, and veins<br>- Circulatory system and the heart	- The Cardiovascular System: The Heart<br>- The Cardiovascular System: Blood Vessels and Circulation	12[a],12[b],11[c]	73[a],61[b],51[c]	2
2	Respiratory	- Meet the lungs<br>- People and plants<br>- The bronchial tree<br>- Inhaling and exhaling<br>- How does lung volume change?<br>- Thermoregulation in the lungs<br>- The lungs and pulmonary system	- The Respiratory System<br>- Fluid, Electrolyte, and Acid-Base Balance	13[a],14[b],12[c]	71[a],59[b],65[c]	1
3	Urinary	- Meet the kidneys!<br>- Kidney function and anatomy<br>- Glomerular filtration in the nephron<br>- Changing glomerular filtration rate<br>- Countercurrent multiplication in the kidney<br>- Urination<br>- The kidney and nephron<br>- Secondary active transport in the nephron	- The Urinary System	8[a],8[b],8[c]	68[a],67[b],54[c]	1
4	Digestive	- Meet the gastrointestinal tract!<br>- Mouth<br>- Teeth<br>- Esophagus<br>- Stomach<br>- Small intestine 1: Structure<br>- Small intestine 2: Digestion<br>- Small intestine 3: Absorption<br>- Liver<br>- Hepatic lobule<br>- Biliary tree<br>- Exocrine pancreas<br>- Endocrine pancreas<br>- Colon, rectum, and anus<br>- Control of the GI tract	- The Digestive System<br>- Metabolism and Nutrition	13[a],13[b],12[c]	61[a],49[b],36[c]	2
5	Hematologic	- What's inside of blood?<br>- Red blood cells<br>- Blood types<br>- Blood cell lineages<br>- Life and times of RBCs and platelets<br>- Hemoglobin<br>- Hemoglobin moves O2 and CO2<br>- Fetal hemoglobin and hematocrit<br>- Oxygen content<br>- How do we make blood clots?<br>- Coagulation cascade<br>- Bohr effect vs. Haldane effect	- The Cardiovascular System: Blood	10[a],36[b+c]	74[a],44[b+c]	1
6	Immune	- Role of phagocytes in innate or nonspecific immunity<br>- Types of immune responses: Innate and adaptive, humoral vs. cell-mediated<br>- B lymphocytes (B cells)<br>- Professional antigen presenting cells (APC) and MHC II complexes<br>- Helper T cells<br>- Cytotoxic T cells<br>- Review of B cells, CD4+ T cells and CD8+ T cells<br>- Clonal selection<br>- Self vs. non-self immunity<br>- How white blood cells move around<br>- Inflammatory response<br>- Blood cell lineages	- The Lymphatic and Immune System	10[a],10[b+c]	78[a],48[b+c]	1
7	Nervous	- Introduction to neural cell types<br>- Anatomy of a neuron<br>- Overview of neuron structure<br>- Overview of neuron function<br>- Sodium-potassium pump<br>- Correction to sodium-potassium pump video<br>- Electrotonic and action potentials<br>- Saltatory conduction in neurons<br>- Synapse structure<br>- Neuronal synapses (chemical)	- The Nervous System and Nervous Tissue<br>- Anatomy of the Nervous System<br>- The Somatic Nervous System<br>- The Autonomic Nervous System<br>- The Neurological Exam	16[a],16[b],16[c]	57[a],47[b],49[c]	3

		- Types of neurotransmitters<br>- Types of neurotransmitter receptors<br>- Structure of the nervous system About Transcript<br>- Functions of the nervous system<br>- Motor unit<br>- Peripheral somatosensation<br>- Muscle stretch reflex<br>- Autonomic nervous system<br>- Upper motor neurons<br>- Somatosensory tracts<br>- Cerebral cortex				
8	Muscular	- Myosin and actin<br>- How tropomyosin and troponin regulate muscle contraction<br>- Role of the sarcoplasmic reticulum in muscle cells<br>- Anatomy of a skeletal muscle cell<br>- Three types of muscle<br>- Motor neurons<br>- Neuromuscular junction, motor end-plate<br>- Type 1 and type 2 muscle fibers<br>- Calcium puts myosin to work<br>- Muscle innervation<br>- Autonomic vs somatic nervous system<br>- Thermoregulation by muscles	- The Muscular System<br>- Muscle Tissue	18[a],20[b],19[c]	69[a],55[b],49[c]	1
9	Skeletal	- Skeletal structure and function<br>- Microscopic structure of bone - the Haversian system<br>- Cellular structure of bone<br>- Skeletal endocrine control<br>- Cartilage<br>- Ligaments, tendons, and joints	- Bone Tissue and the Skeletal System<br>- Joints<br>- The Appendicular Skeleton<br>- Axial Skeleton	10[a+b+c]	43[a+b+c]	0.5
10	Endocrine	- Endocrine gland hormone review<br>- The hypothalamus and pituitary gland<br>- Hormone concentration metabolism and negative feedback<br>- Types of hormones<br>- Cellular mechanism of hormone action	- The Endocrine System	10[a],10[b+c]	47[a],55[b+c]	0.5
11	Integumentary	- Meet the skin!<br>- What is skin?<br>- What lies beneath the epidermis? (Dermis and Hypodermis)<br>- Where do our nails and hair come from?<br>- What's in sweat? (Holocrine, Apocrine, Merocrine Glands)<br>- LeBron Asks: Why does sweating cool you down?<br>- Overview of Sensation and Meissner's Corpuscle<br>- Pacinian's Corpuscle and Merkel's Disk<br>- Ruffini's Ending and Hair Follicle Receptor<br>- Pain and temperature<br>- Thermoregulation by muscles	- The Integumentary System<br>- The Tissue Level of Organization<br>- The Cellular Level of Organization	10[a+b+c]	36[a+b+c]	1
12	Lymphatic	- Why we need a lymphatic system<br>- How lymphatic vessels move fluid<br>- The lymphatic system's role in immunity<br>- Lipid and protein transport in the lymphatic system<br>- What is actually in lymph	- The Lymphatic and Immune System	10[a+b+c]	57[a+b+c]	1
13	Reproductive	- Welcome to the reproductive system<br>- Anatomy of the male reproductive system<br>- Transport of sperm via erection and ejaculation<br>- Spermatogenesis<br>- Testosterone<br>- Basics of egg development<br>- The ovarian cycle<br>- Meet the placenta!<br>- Reproductive cycle graph - Follicular phase<br>- Reproductive cycle graph - Luteal phase<br>- Estrogen<br>- Maternal changes in pregnancy<br>- Labor (parturition)<br>- Breast anatomy and lactation	- The Reproductive System<br>- Development and Inheritance	10[a],10[b]	40[a],39[b]	1

[a] Difficulty level easy
[b] Difficulty level medium
[c] Difficulty level hard
[+] Combination of levels
