# Supplementary material for: Basic Medical Training for Refugees via Collaborative Blended Learning: Quasi-Experimental Design
Source: J Med Internet Res. 2021 Mar 24;23(3):e22345. doi: 10.2196/22345 (PMC8074855; doi:10.2196/22345)
Supplement: Multimedia Appendix 3 [file jmir_v23i3e22345_app3.pdf]

Multimedia Appendix 3. The complete characteristics of the intervention and control group participants.

Students [ID]	Written score [%]	Oral score [%]	Origins	Sexe	Prior knowledge	Absolute frequency
K1[a]	75 (56/75)	100 (20/20)	South Sudan	Male	Yes	Origins: 2[d],1[e],3[f],1[g], 3[h],1[i]
K2[a]	54 (41/75)	75 (15/20)	South Sudan	Male	No	Sexe: 3[j],8[k]
K3[a]	71 (53/75)	95 (19/20)	South Sudan	Male	No	Prior knowledge: 4[l],7[m]
K4[a]	42 (32/75)	70 (14/20)	Somalia	Male	No	
K5[a]	45 (34/75)	80 (16/20)	Sudan	Male	No	
K6[a]	36 (27/75)	70 (14/20)	Ethiopia	Female	Yes	
K7[a]	52 (39/75)	45 (9/20)	Burundi	Female	Yes	
K8[a]	17 (13/75)	10 (2/20)	RDC	Female	No	
K9[a]	80 (60/75)	95 (19/20)	Burundi	Male	Yes	
K10[a]	46 (35/75)	40 (8/20)	RDC	Male	No	
K11[a]	39 (29/75)	60 (12/20)	RDC	Male	No	
D1[b]	39[c]	77[c]	NA	NA	NA	Origins: 1[f],17[g]
D2[b]	38[c]	42[c]	NA	NA	NA	Sexe: 5[j],13[k]
D3[b]	48[c]	38[c]	NA	NA	NA	Prior knowledge: 7[l],11[m]
D4[b]	29[c]	45[c]	NA	NA	NA	
D5[b]	28[c]	52[c]	NA	NA	NA	
D6[b]	38[c]	52[c]	NA	NA	NA	
D7[b]	28[c]	47[c]	NA	NA	NA	
D8[b]	37[c]	38[c]	NA	NA	NA	
D9[b]	22[c]	62[c]	NA	NA	NA	
D10[b]	0[c]	30[c]	NA	NA	NA	
D11[b]	8[c]	39[c]	NA	NA	NA	
D12[b]	56[c]	79[c]	NA	NA	NA	
D13[b]	41[c]	50[c]	NA	NA	NA	
D14[b]	7[c]	43[c]	NA	NA	NA	
D15[b]	21[c]	42[c]	NA	NA	NA	
D16[b]	27[c]	53[c]	NA	NA	NA	
D17[b]	48[c]	93[c]	NA	NA	NA	
D18[b]	32[c]	40[c]	NA	NA	NA	

Results

Written score			
		51[n](18.7)[o]; 46[p](11.9)[q]; 17[r]-80[s](63)[t]; 0.1[u], 1.1[v], 5.6[w]	([a])
		30[n](14.9)[o]; 31[p](12.6)[q]; 0[r]-56[s](56)[t]; -0.4[u],-0.7[v],3.5[w]	([b])
By categories			
	Male	57[n](16.3)[o]; 50[p](14.1)[q]; 39[r]-80[s](41)[t]; 0.3[u], -1.9[v], 5.8[w]	([a])
		NA	([b])
	Female	35[n](17.5)[o]; 36[p](23.7)[q]; 17[r]-52[s](35)[t]; -0.06[u], -2.33[v], 10.12[w]	([a])
		NA	([b])
	Prior knowlege:Yes	61[n](20.5)[o]; 64[p](20.8)[q]; 36[r]-80[s](44)[t]; -0.2[u],-2.2[v],10.7[w]	([a])
		NA	([b])
	Prior knowlege:No	45[n](16.3)[o]; 45[p](8.9)[q]; 17[r]-71[s](54)[t]; -0.1[u],-0.8[v],6.2[w]	([a])
		NA	([b])
Oral score			
		67[n](27.2)[o]; 70[p](37)[q]; 10[r]-100[s](90)[t]; -0.6[u],-0.7[v],8.2[w]	([a])
		51[n](16.6)[o]; 46[p](9.6)[q]; 30[r]-93[s](63)[t]; 1.1[u],0.3[v],3.9[w]	([b])
By categories			
	Male	77[n](20.3)[o]; 78[p](26)[q]; 40[r]-100[s](60)[t]; -0.5[u],-1.2[v],7.2[w]	([a])
		NA	([b])
	Female	42[n](30.1)[o]; 45[p](37.1)[q]; 10[r]-70[s](60)[t]; -0.1[u],-2.3[v],17.4[w]	([a])
		NA	([b])
	Prior knowlege:Yes	78[n](25.3)[o]; 83[p](22.2)[q]; 45[r]-100[s](55)[t]; -0.3[u],-2.1[v],12.7[w]	([a])
		NA	([b])
	Prior knowlege:No	61[n](28.4)[o]; 70[p](14.8)[q]; 10[r]-95[s](85)[t]; -0.6[u],-1.1[v],10.7[w]	([a])
		NA	([b])

[a] Intervention Group
[b] Control Group
[c] Absolute number not provided by teachers
[d] Burundi
[e] Ethiopia
[f] Republic democratic congo
[g] Somalia
[h] South Sudan
[i] Sudan
[j] Female
[k] Male
[l] Yes
[m] No
[n] Mean
[o] Standard deviation
[p] Median
[q] Median absolute deviation
[r] Minimum
[s] maximum
[t] Range
[u] Skew
[v] Kurtosis
[w] Standard error
