# Supplementary material for: Basic Medical Training for Refugees via Collaborative Blended Learning: Quasi-Experimental Design
Source: J Med Internet Res. 2021 Mar 24;23(3):e22345. doi: 10.2196/22345 (PMC8074855; doi:10.2196/22345)
Supplement: Multimedia Appendix 4 [file jmir_v23i3e22345_app4.pdf]

Multimedia Appendix 4.

Analysis was performed with the WhatsAnalyzer program provided by the University of Würzburg.

Figure 1. General statistics for WhatsApp Group.

The chat has **21 members**.

In total, **677 messages** were sent.

In total, **148 media messages** were sent. Media messages cover photos, videos, gifs, voice messages, location information and contacts.

The longest message with **1686 characters** was sent by **User1** .
The shortest message with **1 characters** was sent by **User1** .

Figure 2. Messages sent per user on WhatsApp chat.

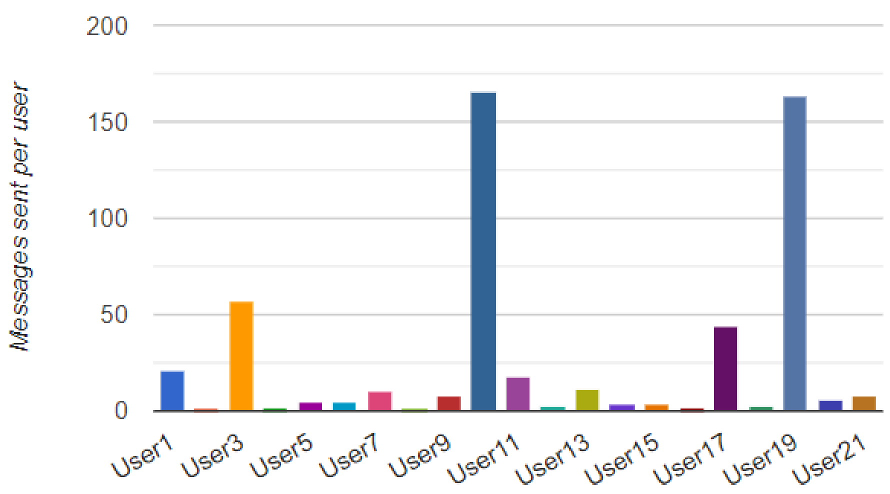

Figure 3. Messages sent on average during Module 1 on the WhatsApp platform.

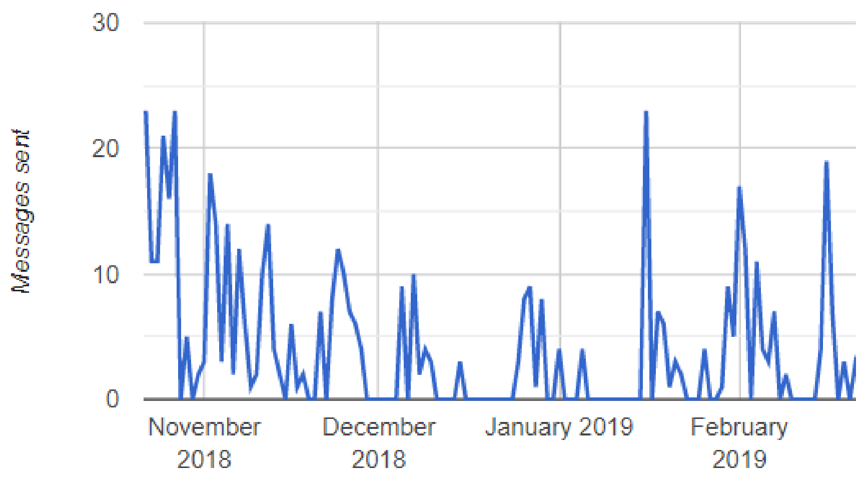

Figure 4. Messages sent on average by days during Module 1 on the WhatsApp platform.

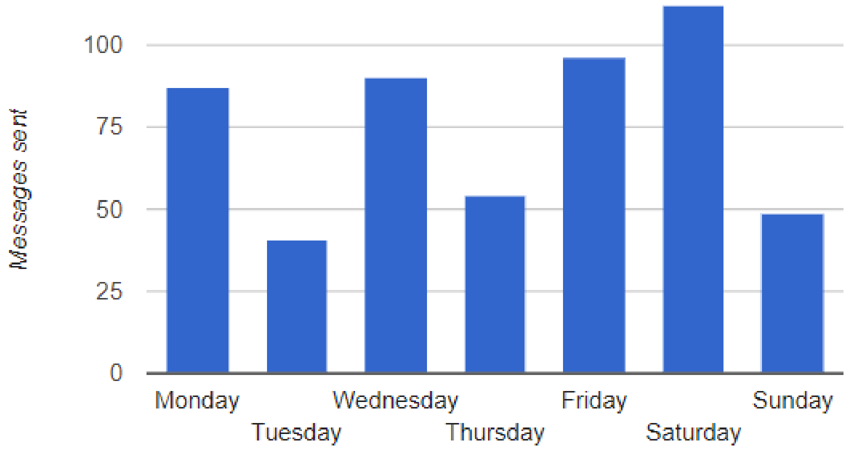

Figure 5. Messages sent on average by hours during Module 1 on the WhatsApp platform.

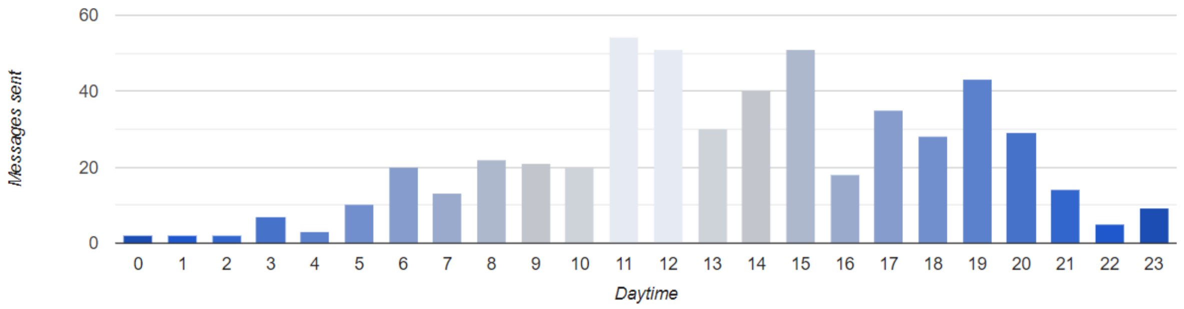

Figure 6. Communication network beetwen users during Module 1.

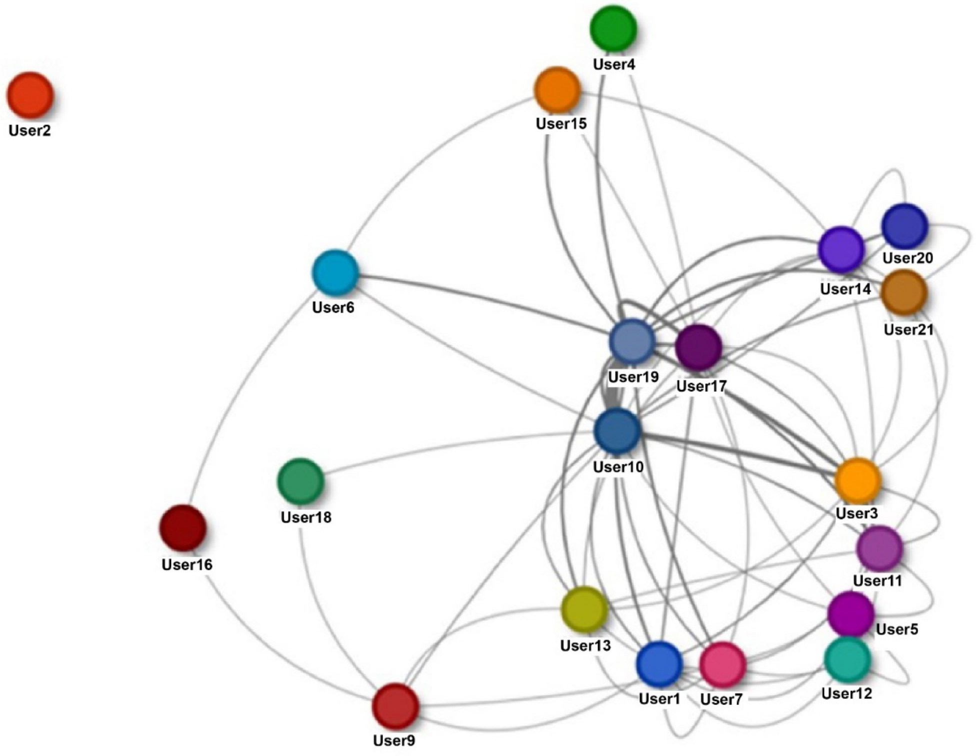

Teachers are User 19 & 21.
