# Supplementary material for: Basic Medical Training for Refugees via Collaborative Blended Learning: Quasi-Experimental Design
Source: J Med Internet Res. 2021 Mar 24;23(3):e22345. doi: 10.2196/22345 (PMC8074855; doi:10.2196/22345)
Supplement: Multimedia Appendix 5 [file jmir_v23i3e22345_app5.pdf]

Multimedia Appendix 5.

List 1. Sentiment analysis on WhatsApp chat by generating the world cloud, fetching sentiment words from text, counting the sentiment words by category, printing sentiment plot.

```
Word frequency TOP 20
will             will   99
inzone         inzone   69
can               can   56
thanks         thanks   52
system         system   50
thank           thank   47
cells           cells   35
one               one   32
hello           hello   29
well             well   29
really         really   29
question     question   29
kakuma         kakuma   27
teacher       teacher   27
good             good   24
course         course   23
start           start   23
questions questions   23
now               now   22
use               use   22
```

```
Sentiment count
1         anger    48
2  anticipation   249
3       disgust    39
4          fear    98
5           joy   149
6       sadness    73
7      surprise    69
8         trust   344
9      negative   146
10     positive   516
Worldcloud Whatsapp Group
```

Figure 1. Text mining word cloud on WhatsApp chat.

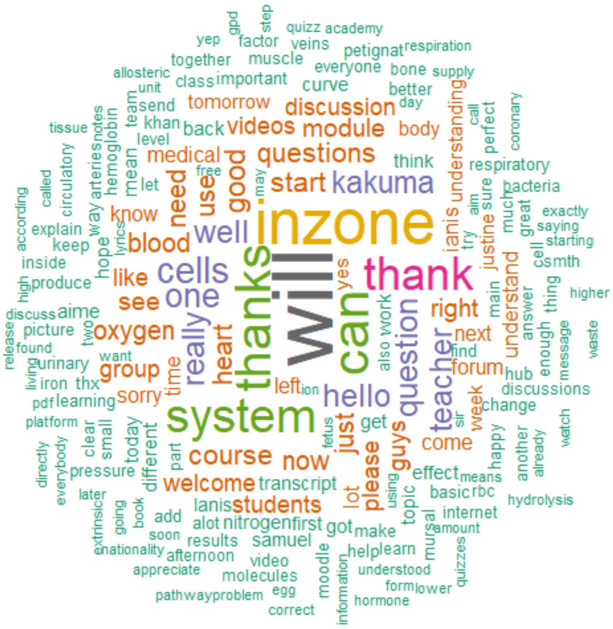

Figure 2. Barplot representation of the total sentiment score in the WhatsApp group.

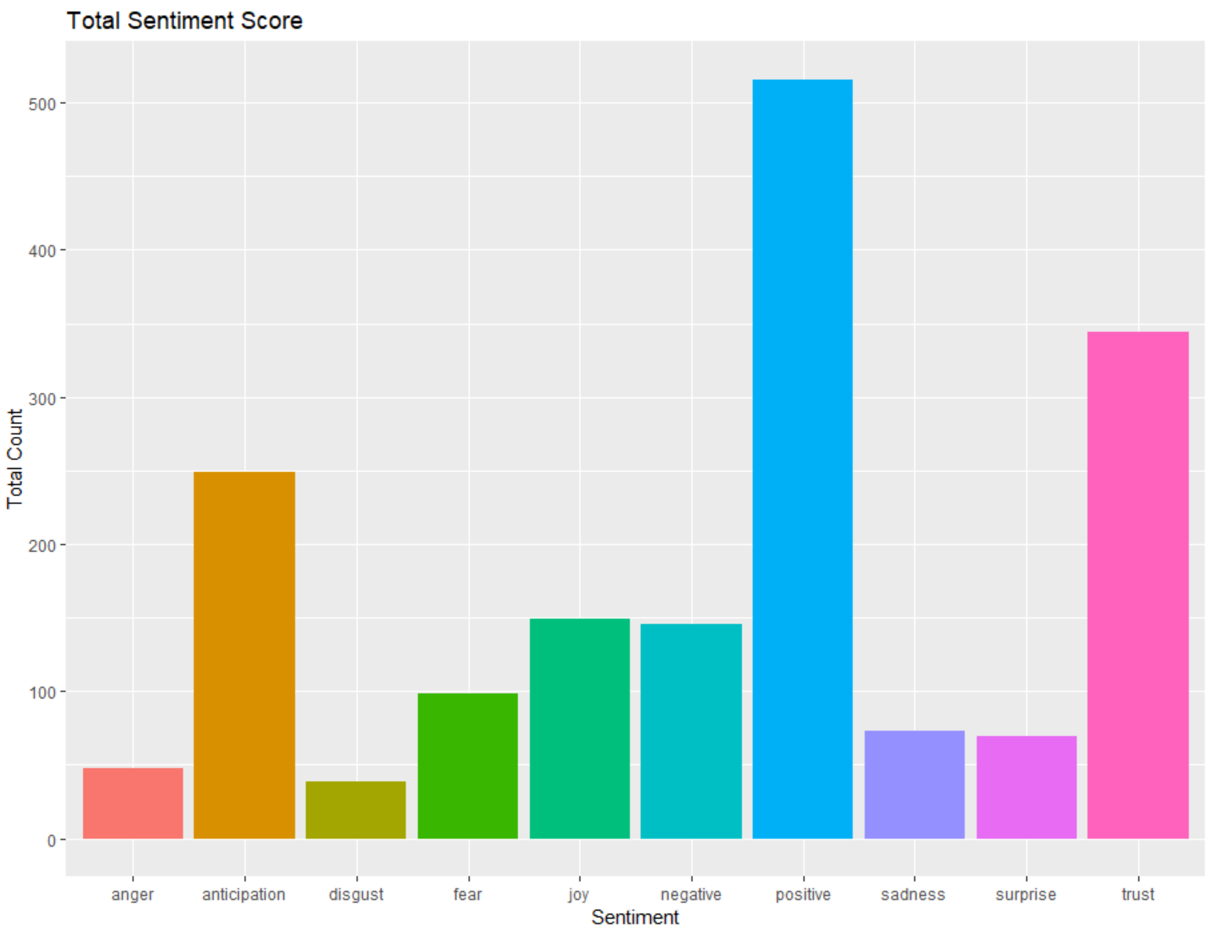

Text mining graphical representation of sentiment analysis on 10 different sentiments. They are represented on the x-axis and on the y-axis the absolute frequency of each sentiment. The graph shows that the most representative sentiment was positive (n=516), followed by trust (n=344) and the least representative was disgust (n=39).
